# Basal Septal Hypertrophy as the Early Imaging Biomarker for Adaptive Phase of Remodeling Prior to Heart Failure

**DOI:** 10.3390/jcm11010075

**Published:** 2021-12-24

**Authors:** Fatih Yalçin, Hulya Yalçin, Nagehan Küçükler, Serbay Arslan, Oguz Akkuş, Alparslan Kurtul, Maria Roselle Abraham

**Affiliations:** 1 Cardiology UCSF Health, Department of Medicine, University of California at San Francisco, San Francisco, CA 94143, USA; hzafer44@hotmail.com (H.Y.); roselle.abraham@ucsf.edu (M.R.A.); 2 Department of Cardiology, Mustafa Kemal University, Antakya 31100, Turkey; drnagehan@gmil.com (N.K.); serbayarslan1@gmail.com (S.A.); oakkusfb@gmail.com (O.A.); alpkurtul@yahoo.com (A.K.)

**Keywords:** basal septal hypertrophy, early imaging biomarker, heart failure, hypertension and stressed heart morphology

## Abstract

Hypertension plays a dominant role in the development of left ventricular (LV) remodeling and heart failure, in addition to being the main risk factor for coronary artery disease. In this review, we focus on the focal geometric and functional tissue aspects of the LV septal base, since basal septal hypertrophy (BSH), as the early imaging biomarker of LV remodeling due to hypertensive heart disease, is detected in cross-sectional clinic studies. In addition, the validation of BSH by animal studies using third generation microimaging and relevant clinical observations are also discussed in the report. Finally, an evaluation of both human and animal quantitative imaging studies and the importance of combined cardiac imaging methods and stress-induction in the separation of adaptive and maladaptive phases of the LV remodeling are pointed out. As a result, BSH, as the early imaging biomarker and quantitative follow-up of functional analysis in hypertension, could possibly contribute to early treatment in a timely fashion in the prevention of hypertensive disease progression to heart failure. A variety of stress stimuli in etiopathogenesis and the difficulty of diagnosing pure hemodynamic overload mediated BSH lead to an absence of the certain prevalence of this particular finding in the population.

## 1. Background

Heart failure (HF) is a progressive process and associated with the left ventricular (LV) remodeling [1]. Hypertension is the major risk factor for cardiovascular disease and associated with pressure-overload, which increases LV wall stress and ultimately leads to heart failure [2,3]. Optimal antihypertensive medication reduces LV myocardial mass and slows the progression of hypertensive LV remodeling to heart failure [4,5]. Real-time three-dimensional echocardiography has documented a non-uniform LV myocardial geometry and a narrowed cavity volume at the LV base in patients with hypertension-mediated global LV remodeling [6,7]. The early determination of hypertensive heart disease using biomarkers possibly contributes to early medical management at the early stage of the disease as prehypertension and could be beneficial in the prevention of the progressive LV remodeling to heart failure [8,9].

## 2. Basal Septal Hypertrophy and Clinic Observations

It is known that LV segmental remodeling could be the case in early-stage hypertension described as borderline hypertension, and the septal wall is speculated to be the first LV wall to become hypertrophic in clinical practice according to cross-sectional studies [10,11,12]. Increased myocardial dynamics was suggested to be related to focal hypertrophy. In addition to this non-uniform LV geometry with segmental hypertrophy of septal base (basal septal hypertrophy, BSH), myocardial hypercontractility is determined in imaging reports as a usual finding in the early stage of hypertensive heart disease [13,14]. We confirmed hyperdynamic septal basal tissue quantitatively by the combined analysis of tissue Doppler imaging and dobutamine stress echocardiography in BSH [15]. However, the determination of hypertension under stress in some symptomatic patients with dyspnea and hyperdynamic function is not recalled during examination and technic analysis [16]. Therefore, we remarked the importance of investigation for hypertension and technic analysis with ultrasound in symptomatic patients with hyperdynamic LV function [17].

## 3. BSH and Animal Validation Studies

Our earlier reports showed a high heart rate–pressure product and high LV outflow tract blood flow as well as basal septal hyperdynamic tissue under stress in BSH patients with hypertension [15,18,19,20]. These clinic observations possibly represented a compensatory hemodynamic adaptation to increased stress stimuli in this group of patients. Beyond these cross-sectional observations, we decided to determine the progression of LV segmental remodeling prospectively. We used treadmill exercise for physiologic and transaortic construction for pathologic stress stimuli and third-generation microscopic ultrasonography in small animals for the determination of segmental LV remodeling evolution. In this study, we noted for the first time that BSH is the early imaging biomarker of LV remodeling under both physiologic and pathologic stress [21]. Nevertheless, since we detected BSH development as initial LV remodeling prospectively in an animal model in both physiologic stress-mediated remodeling with normal organization of myocytes and pathologic with myocyte hypertrophy and collagen accumulation, a correct diagnosis for the nature of BSH should be documented by blood pressure (BP) monitorization to explore whether or not pressure-overload is the case.

In the animal validation studies, we also detected a remarkable velocity increment in the early time points after transaortic construction in pathologic stress, which was consistent with the findings in cross-sectional clinic studies [22]. However, there was a sharp transition in the basal septal tissue function after the initiation of the maladaptive phase of LV remodeling under pathologic stress, while LV function was preserved in animals with physiologic stress. Nevertheless, preserved global LV function at the BSH stage with compensatory tissue hyperfunction prior to maladaptation could lead to a diagnostic difficulty in clinical practice if pressure-overload is not completely eliminated. On the other hand, previous ultrasonographic data regarding BSH in the last two decades [15,18,19,20] have recently been confirmed by cardiac magnetic resonance (CMR) studies in healthy individuals with initial septal remodeling.

BP monitorization under stress is insufficient in patients with LV septal hypertrophy during clinical practice. In fact, no attempt was made for BP recording during exercise even in the comprehensively planned CMR study in which physiologic exercise in a healthy population was speculated to be the reason for septal hypertrophy [23]. We emphasized the importance of BP monitoring in imaging studies on initial septal remodeling, since physiologic septal remodeling cannot be proven without the complete elimination of hemodynamic overload due to increased BP at stress [24]. Furthermore, we have recently considered reportingandon hemodynamic overload using BP recording during exercise in healthy people or even endurance athletes with septal hypertrophy determined using CMR [25] and the importance of BSH as the early imaging biomarker in the prevention of global remodeling by an effective medical management [26].

### Striking Difference between Animal and Human BSH Data

BSH plays a role as a common denominator for variety of stress stimuli beyond basic hemodynamic stress-mediated LV remodeling. Due to this complex nature of BSH, we previously described this morphological finding as the ‘stressed heart morphology’ (SHM), which was documented as an imaging figure in both acute and chronic stress-mediated heart conditions such as acute stress cardiomyopathy [27] and hypertension [26]. Despite the fact that hemodynamic overload seems to be a unique pathophysiologic mechanism for LV global remodeling, the LV base represents an extremely sensitive feature under different types of stress stimuli, since it is associated with predominant sympathetic innervation.

**Figure 1:**
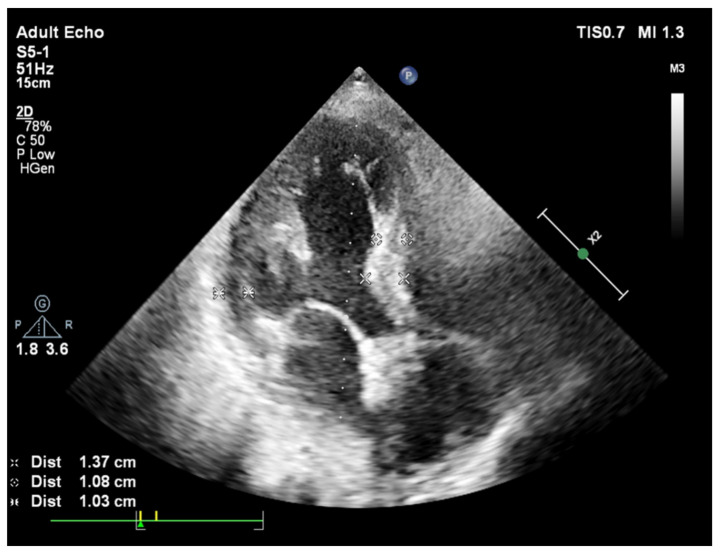
A slight curve of septal base from apical 4 chamber view during end-diastole in a patient with systemic hypertension and basal septal hypertrophy.

**Figure 2:**
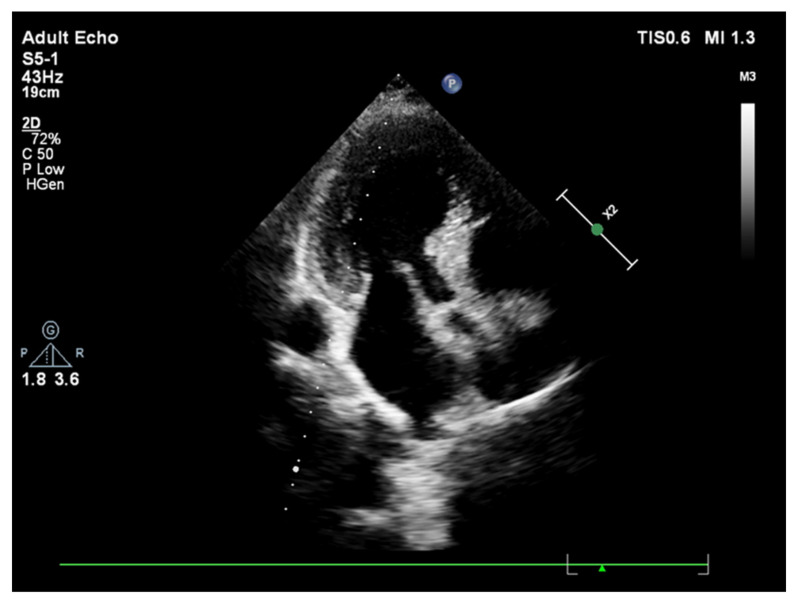
A sharp curvature of more remarkable basal septal hypertrophy from apical 4 chamber view during end-diastole in another hypertensive patient.

**Figure 3:**
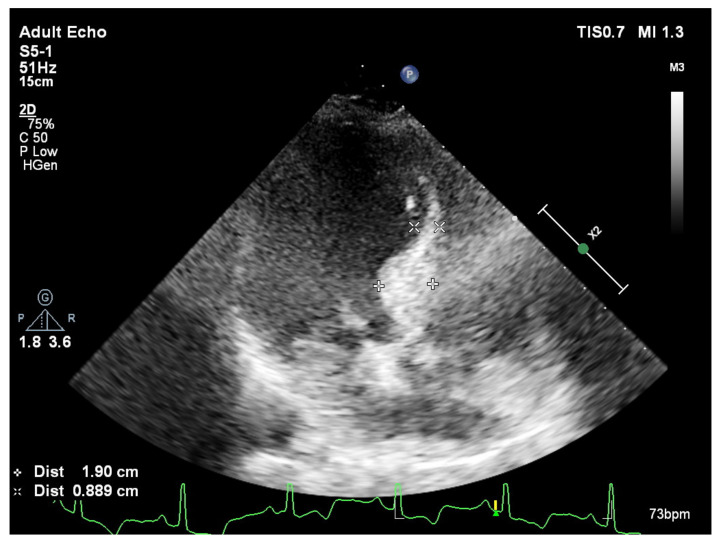
Predominantly placed hypertrophy over septal base from apical 4 chamber view during end-diastole in a patient with aortic stenosis and basal septal hypertrophy.

In fact, while a smooth curvature is the case in some patients (Figure 1), another group of hypertensive patients could have extremely predominant BSH without any remodeled segments over the rest of the LV (Figure 2). We have recently described the mechanical component of BSH in aortic stenosis (Figure 3) in addition to the other etiologies including emotional in acute stress cardiomyopathy and functional due to an increased afterload in hypertensive patients [28]. All these different mechanisms of stress induction in humans may explain a more complex nature for the development of this morphology. SHM could represent an individually variable heterogenous distribution of myocardial remodeling in humans and this heterogeneity supports the potential influence of additional mental or emotional stress-mediated contributors in humans.

Sympathetic overdrive more dominantly involves the LV base compared to the midapical myocardial segments. However, despite the fact that it is a potentially possible etiologic mechanism, there has not been any chronic mental or emotional disorder documented or described except acute stress cardiomyopathy in the development of SHM. Heterogeneity involved in remodeling in humans produces a dilemma not only in a certain diagnosis of pure hemodynamic overload-mediated BSH in hypertensive patients, but in the certain determination of BSH prevalence in the population.

**Figure 4:**
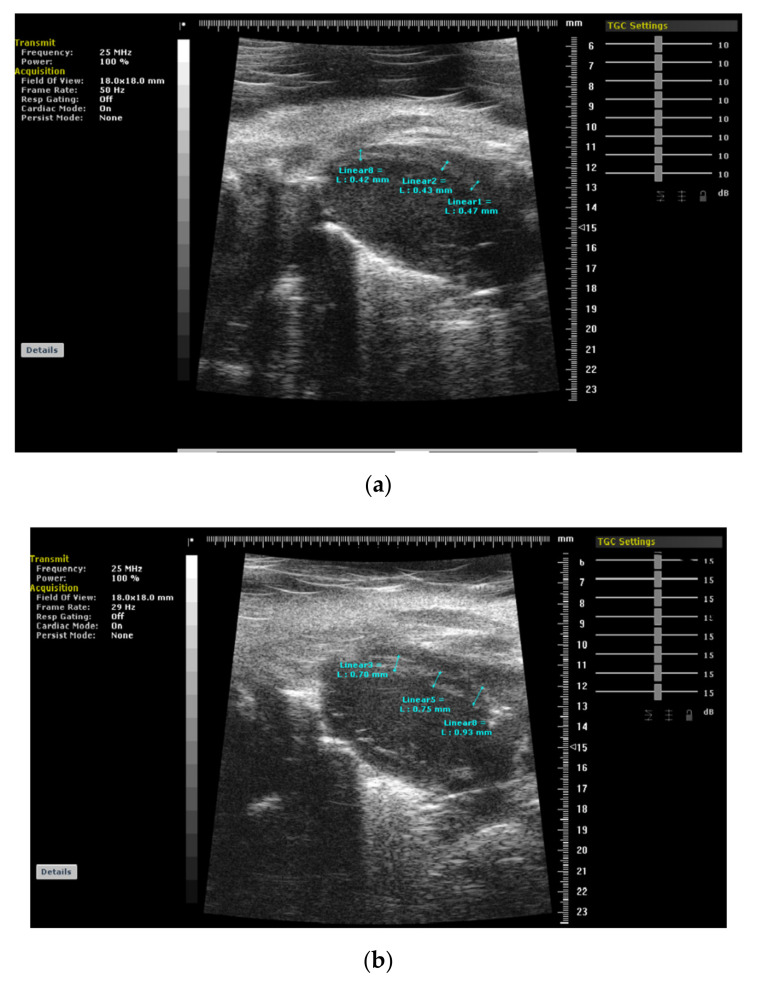
(**a**,**b**): Cardiac images of a mice using 3rd generation microscopic ultrasound show normal cardiac geometry and a regularly remodeled septal wall with thickerseptal base at 4 weeks after stress induction due to pressure-overload (TAC: transverse aortic construction), respectively.

On the other hand, years later, we have determined LV segmental remodeling in an animal model that is associated with a very regular distribution (Figure 4a,b) under stress-mediated hemodynamic overload differently from an individually variable heterogeneity and focally predominant BSH in human data. Third-generation microscopic ultrasonography provided a unique opportunity for us to document the regional evolution of LV remodeling prospectively. Microimaging also confirmed previous cross-sectional clinic studies in hypertension that support BSH as the first remodeled region that we validated as the early imaging biomarker in animal validation studies [21,22]. Recently, we suggested that scientists should be cautious in the quantification of this morphologic finding, because it is possible to detect an extremely large spectrum of BSH heterogeneity in clinical practice [29,30].

## 4. Heart Failure with Preserved Ejection Fraction

HF with preserved ejection fraction (HFPEF) is a relatively new clinical entity and the number of HFPEF patients is increasing worldwide. As the majority of the HFPEF patients have systemic hypertension, HFPEF is usually accepted as a hypertension-mediated phenomenon. The preserved global systolic function has been documented by freehand three-dimensional echocardiography at rest in HFPEFpatients [31]. However, despite a normal global EF, contractile abnormalities are more common in HFPEF, unlike patients with hypertensive global LV remodeling [32]. We pointed out the importance of the evaluation of contractile reserve by combined diagnostic techniques with stress induction, which may be beneficial for the monitorization of disease progression from an early to an advanced stage and a more effective treatment of hypertensive heart disease [33]. In fact, fibrotic tissue accumulation progressively decreases diastolic filling and results in impaired LV contractility in HFPEF [34]. As both conditions have preserved global EF at rest, it is crucial to separate hypertensive LV remodeling from the HFPEF. It was pointed out that myocardial fibrosis detected using CMR could be beneficial in the detection of patients with HFPEF [35].

## 5. Effective Medical Approach in Hypertensive Disease

The efficacy of preventive measures to slow the progression of hypertensive heart disease supports the concept that hypertrophy is possibly a reversible stage of LV remodeling [5]. However, echocardiographic and nuclear studies document that LV hypocontractility develops with the progression of hypertensive heart disease and leads to an increased risk of cardiovascular events [36,37]. Nevertheless, early medication is crucial, since improvement in midwall or endocardial wall fractional shortening by aggressive antihypertensive treatment is associated with reduced cardiovascular events and heart failure incidence in hypertensive global LV remodeling [5]. As clearly shown in this study, effective antihypertensive therapy is essential to improve LV function and better outcomes. Some observations on apoptosis and fibrotic tissue accumulation were also reported in this group of patients [38,39]. Effective therapy possibly keeps global myocardial function preserved, which was confirmed by tissue Doppler imaging or mitral annular motion using real-time three-dimensional echocardiography that reflects global LV function [40,41]. Preserved cardiac energy metabolism was another consistent finding in the pressure-overload model before the development of heart failure in Dahl salt-sensitive rats [42]. Moreover, even we use the terminology of global LV remodeling for advance cases with hypertensive heart disease; we detected predominant septal geometry and more severe septal dysfunction than free wall in this group and speculated that the early involvement of the septum by BSH could possibly be a reason for more severe regional involvement in the LV septum with progression of the disease process [43].

Hypertension-mediated HF describes a mild LV remodeling mostly observed in female patients [44]. It is evident that the majority of these patients have systemic hypertension and preserved global LV systolic function documented usingthree-dimensional echocardiography [31]. Despite a normal global EF, it is emphasized that contractile abnormalities and severe diastolic dysfunction are more common in the HFPEF patients than the patients with hypertensive LVH [45]. In the disease course of hypertension toward the development of HF, prevention of LVH is the main objective in developing novel antihypertensive therapy. Defining the severity of LV remodeling may help clarify pathophysiology and define the success of medical management in the target HFPEF population [46]. It was documented in an I-Preserve study that the elderly HFPEF patients with appropriate medical management are associated with a limited severity of LVH, which is not an ideal target for new therapeutic options [47].

It has been mentioned that myocardial fibrosis is associated with diastolic HF in hypertensive heart disease [48]. The accumulation of fibrotic tissue leads to a decrease in the compliance of cardiac anatomy and directly impairs diastolic filling, ultimately affecting LV contractility in HFPEF [34]. The lack of an effective therapeutic regimen for elderly HFPEF patients may be explained by the level of myocardial fibrosis and its consequences [49]. Hypertensive LVH and HFPEF both have preserved global EF at rest. Hence, it is crucial to diagnose hypertensive LVH and HFPEF accurately in order to appropriately manage the individual patient. In hypertensive patients, an evaluation of contractile reserve may be beneficial in monitoring the progression of the disease to heart failure [50]. Recently, the importance of a quantitative assessment of contractile reserve has been described for the follow-up of patients with hypertensive heart disease [33].

Age-related myocardial fibrosis may adversely affect the contractile reserve, especially in elderly hypertensive individuals. Recent data have shown that contractility may become blunted by stress induction in patients older than 70 [32]. Interestingly, the quantitative evaluation of contractile reserve under stress in younger patients with LVH is relatively preserved [15]. This clearly supports the concept that an evaluation of resting echocardiographic indices may not be sufficient for the correct diagnosis and differentiationof hypertensive LVH from HFPEF. It has been reported that the use of angiotensin 1 receptor blocker treatment may reduce the progression of myocardial fibrosis [38,39].

## 6. Cardiovascular Outcomes of Patients with HFPEP Who Are on Effective Antihypertensive Treatment

The collected data showed that blood pressure control is an essential step in patients with hypertension-mediated consequences leading to vascular mortality [9,51]. The Asheville Project has proven that hypertension control improves, and the cardiovascular event rate drops from 77 to 38 per 1000 people by utilizing a community-based pharmacy-directed medication care management program [52]. However, when this preventable disease is not controlled by available measures and progresses, it is associated with the predictable adverse outcomes. High mortality, which is similar with systolic HF, as clearly documented by the Olmstead County Study, is possibly due to the very low prevalence of appropriate medical management. In this community-based study, only 17% of the HFPEF patients were on angiotensin-converting enzyme inhibitor therapy [53].

According to another comprehensive study from the Johns Hopkins Medical Institutions, HFPEF patients who are on an effective medical therapeutic regimen (68% use of an ACE inhibitor) have similar endocardial and mid-wall fractional shortening compared to that of hypertensive LVH [46]. This promising comprehensive functional finding in HFPEF patients who are on appropriate medications has raised the possibility that the community-based finding of high mortality in patients with HFPEF may not reflect the true natural course of the disease. In fact, an Epi-Cardio Survey from Argentina has shown that in-hospital HFPEF patients on ACE inhibitors or AT1 receptor blockers (greater than 60%) have a lower mortality than that of systolic HF patients with similar comorbidities who are on appropriate medical management [54]. These observations may confirm the importance of early and optimal medical management in the survival of patients with hypertensive heart disease.

Atrial fibrillation (AF) is an important problem during follow-up in hypertensive patients. HFPEF with AF is associated with a greater mean LV mass compared to systolic HF patients with AF, which supports the special importance of long-standing hypertension as the risk factor in HFPEF patients [55]. It may appear that patients with HF, but normal or nearly normal systolic function, have much better prognosis [56]. Similar to the patients with HFPEF and sinus rhythm, Badheka et al. [57] have documented that the patients with HFPEF and AF are associated with better survival compared to systolic HF patients with AF. It is also known that HFPEF patients are less likely to be in a higher NYHA class, [58] have a better quality of life, [59] and lower hospital admission rates [60] than those with systolic HF.

As a result, since LV remodeling leading to HF is a progressive process, an LV segmental evaluation seems important, not only in the determination of BSH as the early imaging biomarker, but in a severity assessment of LV myocardial regions in advance hypertensive cases with global LV remodeling. It is documented in the literature that precise patientmonitorization using novel cardiac imaging can successfully contribute to differentiating the stages of disease course and successful, widespread long-term medical management.
